# Living with Tumour necrosis factor receptor-associated periodic fever syndrome (TRAPS)

**DOI:** 10.1186/1546-0096-13-S1-P23

**Published:** 2015-09-28

**Authors:** P Dandekar, J Gregson, R Campbell, F Bianic

**Affiliations:** 1 Novartis Pharma AG, Basel, Switzerland; 2 MAPI, Uxbridge, UK; 3 MAPI, Nanterre, France

## Introduction

TRAPS is a genetic disorder characterised by recurrent attacks of fever, abdominal and muscle pain and rash, most common in patients of northern European origin.^1^ Very little is known about the patient experience with TRAPS.

## Objectives

To qualitatively assess the burden of disease on patients and caregivers and describe patient's journey.

## Patients and methods

TRAPS patients or caregivers (paediatric patients) (N=16) were recruited via rare disease experts and patient groups. A 20 page pre-interview questionnaire and in-depth 90 minute interview were completed. Complete data (n=15) were quantified with topics focused on symptoms, diagnosis, burden, treatment experience and unmet needs.

## Results

Most patients were female (n=13) and had a family history of TRAPS (n=9). Physical functioning was greatly impaired during an attack with most common symptoms including fever, abdominal pain, joint pain, rash, vomiting and diarrhoea. Fever was the most prominent symptom in children and muscle and joint pain in adults. Paediatric patients reported missing school due to attacks or attending medical appointments were common. Adults frequently missed work for similar reasons, resulting in anxiety over work due to frequent absences. Damage to education or career had a negative financial impact as did travelling long distances to medical appointments. Social activities were also restricted, creating a sense of isolation, embarrassment and uncertainty. Delay in diagnosis was frequently reported with time to diagnosis greater than five years in more than 50% of patients. Steroids are typically the first treatment, while providing temporary relief may lead to uncertainly of long-term side effects, Biologics, typically IL-1 or TNF-alphas, are initiated subsequently and majority of patients reported a decrease in severity and frequency of attacks. Common concerns include, injection site reactions, increased susceptibility to infection, and maintenance of efficacy. Patients expressed interest in alternative administration and storage conditions as potential ways to ease the burden of management. Finally, an increase in disease awareness was important, as many patients receive little information about their condition and are often required to do their own research, perhaps due to lack of physician awareness.

## Conclusions

The study demonstrated that the burden of TRAPS is considerable, impacting physical, social, emotional and practical/financial aspects of patients’ and caregivers lives. Greater physician disease awareness may lead to improvements in diagnosis. Finally, improved therapeutic options with alternative modes of administration are needed for the treatment of TRAPS.
